# Punning

**Published:** 1857-07

**Authors:** 


### PUNNING.

OUR shoemaker is an inveterate punster. In a recent note to us, he says:

"I send the articles requested, and will take pay in journals. I hope they will *last* long, and that there will be no *end* to the wear of them; but whether so or not, I intend to stick to you and your journal like a *plaster*."

One who can pun so well, not only on the technicalities of his own calling, but on those of one of the learned professions, and who has the intelligence to so fully appreciate HALL'S JOURNAL OF HEALTH, must be a practical, finished, and philosophical workman, and we should judge that the boots and shoes of Mr. J. B. Miller, of three hundred and eighty-seven Canal street, New York, will keep out dampness and disease rather better than any others.

